# Recent advances on graphyne and its family members as membrane materials for water purification and desalination

**DOI:** 10.3389/fchem.2023.1125625

**Published:** 2023-01-20

**Authors:** Kayode Hassan Lasisi, Olusegun K. Abass, Kaisong Zhang, Temitope Fausat Ajibade, Fidelis Odedishemi Ajibade, John O. Ojediran, Ehizonomhen Solomon Okonofua, James Rotimi Adewumi, Peter D. Ibikunle

**Affiliations:** ^1^ Key Laboratory of Urban Pollutant Conversion, Institute of Urban Environment, Chinese Academy of Sciences, Xiamen, China; ^2^ Department of Civil Engineering, and ReNEWACT Laboratory, Landmark University, Omu-Aran, Kwara State, Nigeria; ^3^ Key Laboratory of Marine Environment and Ecology, Ministry of Education, Ocean University of China, Qingdao, China; ^4^ Department of Civil and Environmental Engineering, Federal University of Technology, Akure, Nigeria; ^5^ Department of Agricultural and Biosystems Engineering, Landmark University, Omu-Aran, Kwara State, Nigeria; ^6^ Department of Geomatics, University of Benin, Benin City, Nigeria

**Keywords:** GFM_S_, 2D materials, permeabiilty, membrane, computational analysis and strategies, selectivity, graphyne

## Abstract

Graphyne and its family members (GFMs) are allotropes of carbon (a class of 2D materials) having unique properties in form of structures, pores and atom hybridizations. Owing to their unique properties, GFMs have been widely utilized in various practical and theoretical applications. In the past decade, GFMs have received considerable attention in the area of water purification and desalination, especially in theoretical and computational aspects. More recently, GFMs have shown greater prospects in achieving optimal separation performance than the experimentally derived commercial polyamide membranes. In this review, recent theoretical and computational advances made in the GFMs research as it relates to water purification and desalination are summarized. Brief details on the properties of GFMs and the commonly used computational methods were described. More specifically, we systematically reviewed the various computational approaches employed with emphasis on the predicted permeability and selectivity of the GFM membranes. Finally, the current challenges limiting their large-scale practical applications coupled with the possible research directions for overcoming the challenges are proposed.

## 1 Introduction

The importance of water to lives and livelihood cannot be overemphasized. It is not only essential for life but it also constitute fundamental human rights of humanity. Despite its abundant availability on the earth making up to 71% of the universe, only a minute percentage of about 2.5% is available as freshwater for human consumption (Tan et al., 2020). Unfortunately, little or no access to this freshwater is gained for immediate consumption as most are trapped in glaciers and snowfields, and this ultimately results in insufficient potable water also known as water scarcity (Ahmed et al., 2020; Tan et al., 2020). This water stress condition will continue because of the increasing world population, variations in climatic condition, urbanization in conjunction with improved living standards as projected by UN and others (Gude, 2017; WWAP, 2018; United Nations, 2020). Therefore, it is pertinent that new and state-of-the-art technologies are employed to recover water fit for human use from the abundant seawater and wastewater generated by both human and industrial activities. This action will help in conserving and mitigating the future effect of water scarcity. Among many proposed solutions is desalination, a state-of-the-art technology that stems from membrane separation technology. Traditional treatment processes such as screening, filtration, chlorination/fluoridation, coagulation/flocculation, oxidation, and ion exchange (Gregory and Dhond, 1972; Birjandi et al., 2013; Abass et al., 2017; Dos Santos et al., 2017; Cescon and Jiang, 2020; Vecino and Reig, 2022) have been reported as inefficient in treating pollutants such as organic carbon and heavy metals at high levels. Removal of smaller contaminants of ionic size (<5 nm) is equally challenging due to low removal efficiencies, which is mostly achieved at high operating cost (Abu Hasan et al., 2020). On the contrary, nanoporous membrane materials processes such as, electrodialysis, reverse osmosis (RO) and nanofiltration (NF) filters at molecular level. Thus, are able to remove small molecules in form of pollutants from wastewater with removal efficiency up to 95%, at low capital/operating cost with ease of operation (Davenport et al., 2018; Yang et al., 2019; Gurreri et al., 2020; Lasisi and Zhang, 2022).

Largely, RO technique has been employed worldwide over the past decade as a preferred membrane desalination technology over the thermal desalination technologies owing to its higher efficiency and lower energy consumption (Mayor, 2019; Abdullah et al., 2021). Notwithstanding, commonly used polymeric membranes material for RO processes are replete with drawbacks of low water flux and fouling (Heiranian et al., 2015; Mahmoud et al., 2015; Dervin et al., 2016; Kim et al., 2016). However, some techniques have been utilized for the modification of RO membrane to improve its performance. For instance, the preparation of novel membrane materials (Werber et al., 2016; Li et al., 2020a). Novel nanomaterial-based membranes have proven to be uniquely suitable for desalination (Li et al., 2020a), as they allow water molecules to pass through their nanostructures at high rate while limiting the passage of dissolved salts and other solutes (Daer et al., 2015). Some nanomaterials considered as good candidates for desalination membrane include carbon nanotubes (CNTs), zeolites, graphene, and 2D transition-metal carbides, nitrides, and carbonitrides (Mxenes) (Li et al., 2007; Dumée et al., 2010; Gethard et al., 2011; Cohen-Tanugi and Grossman, 2012; Cohen-Tanugi et al., 2016; Karahan et al., 2020). Recent reports show that this new class of membranes possess exceptional flow rate compared to the conventional commercial membranes (Karahan et al., 2020; Ajibade et al., 2021; Wang et al., 2021; Damptey et al., 2022).

**FIGURE 1:**
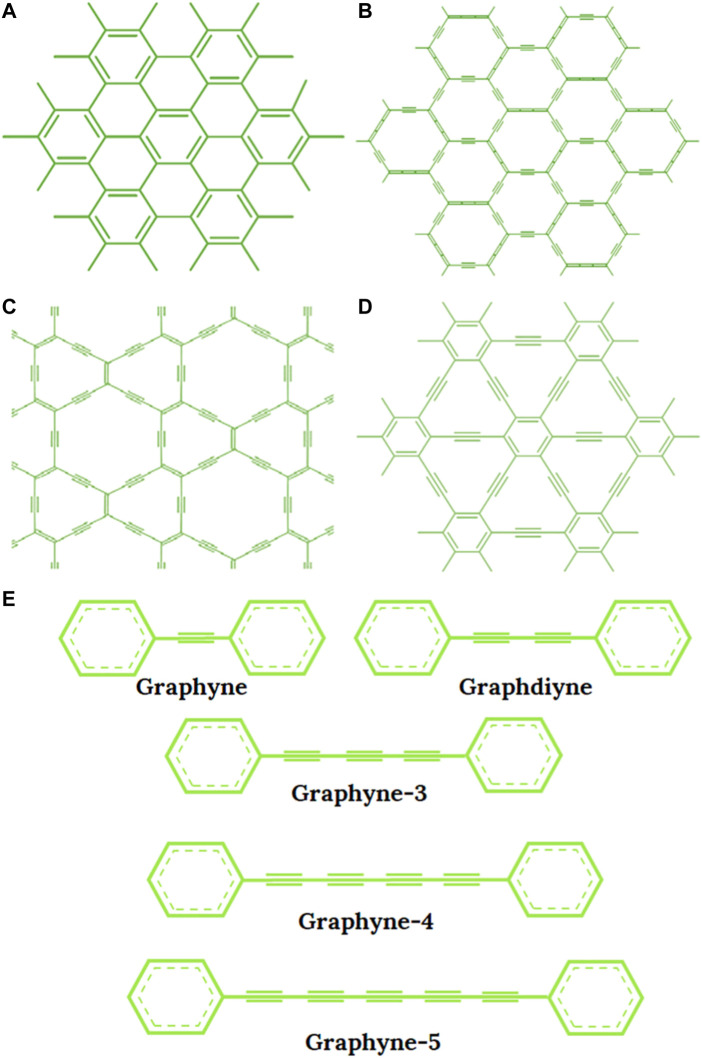
The structure of **(A)** Graphene, **(B)** α-graphyne, **(C)** *β*-graphyne, **(D)** *γ*-graphyne, **(E)** Various types of *γ*-graphyne-n (*n* = 1, 2, 3, 4, and 5).

**FIGURE 2:**
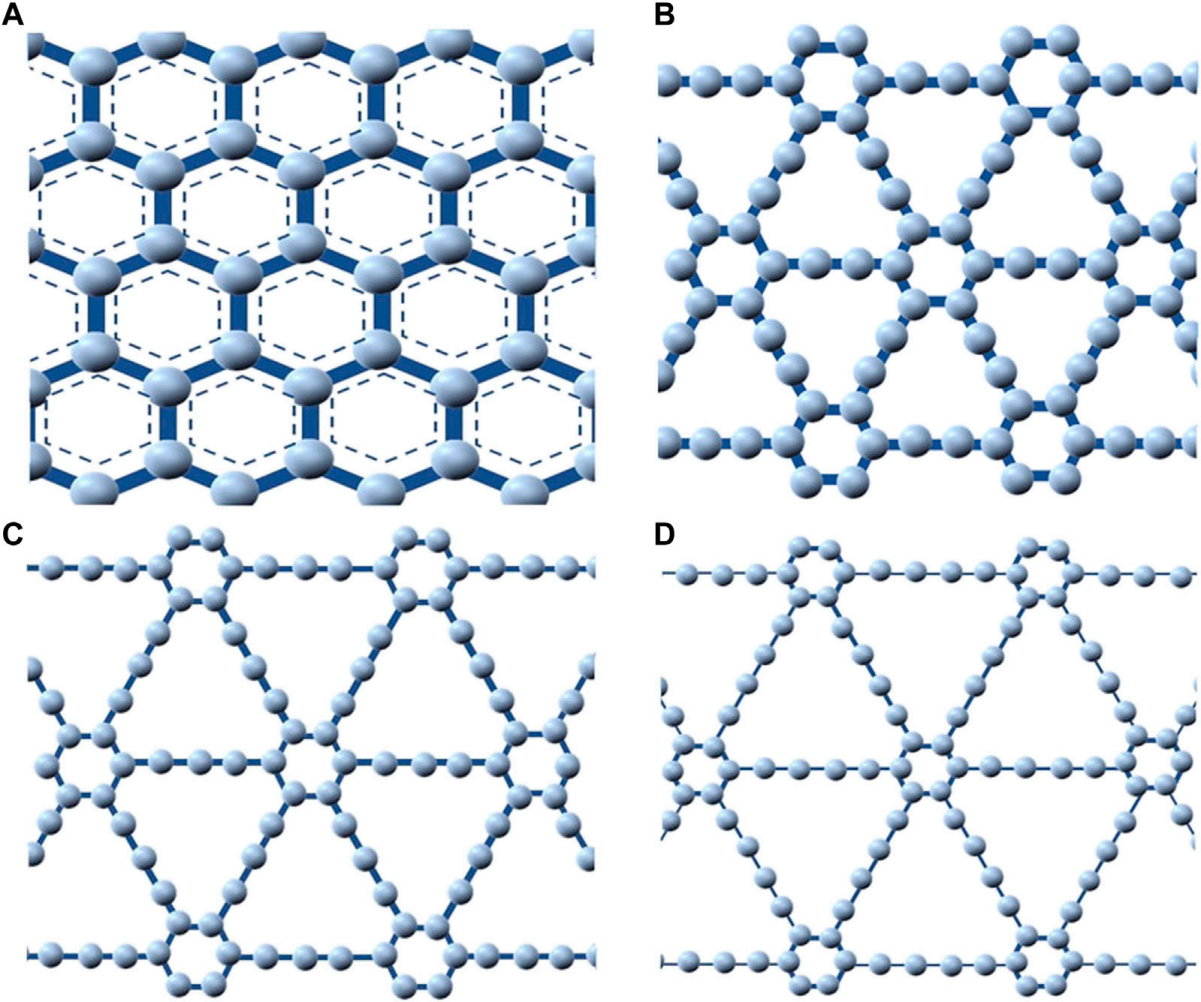
Atomistic structures: **(A)** graphene **(B)** α-graphyne, **(C)** *ß*-graphyne, and **(D)** *γ*-graphyne.

Graphene as an allotrope of carbon has been used extensively as a material in membrane technology for water desalination since patented in 2013 by Lockheed Martin as “Perforene” (Boretti et al., 2018). It has been found to have an atomic thickness, which guarantees high water permeability even better than most commercial NF membranes. In the construction and fabrication of desalination membranes, graphene has been used as pristine graphene, graphene oxide (GO) and in some cases as reduced graphene oxide (rGO) (Perreault et al., 2015). Interestingly, a new family of carbon allotropes called graphyne has recently been discovered as an essential material for effective water desalination. It is a one-atom-thick planar sheet with different forms of extended conjunction between acetylene and benzene groups (Kou et al., 2013; Azamat et al., 2020). In their early predictions, Baughman et al. (1987) described graphyne as stable crystalline carbon allotropes with high degrees of sp hybridization and due to the ethynyl units and aromatic moiety rings (which represents the sp- and sp^2^- hybridized carbon), they were christened after graphite and ethyne (Jia et al., 2017; Mehrdad and Moosavi, 2019). Furthermore, different forms of graphyne were suggested on the strength of inserting triple bonds carbon between benzene rings in graphene including three highly symmetric forms: *α*, *β*, and γ-graphyne as displayed in Figure 1. The graphyne is built on the firm connection of benzene rings by acetylene bonds ─C≡C─ (Figure 1). The pore size can be adjusted by changing the number of acetylene bonds between adjacent benzene rings. This acetylene bond is defined as n and when *n* = 0, the structure becomes graphene but when *n* = 1, 2, 3, …, n, other *γ*-graphyne structures are referred to as the graphyne-n which can be graphyne-1 (or graphyne) graphyne-2 (or graphdiyne) and so on. Figure 2 present the atomistic structures of the main different forms of graphyne for better understanding. In addition, graphyne and its family members (GFMs) have good chemical and mechanical properties and they are chemically inert and stable at room temperature (Enyashin and Ivanovskii, 2011; Cranford et al., 2012). They are also flexible enough to withstand deformation caused by high water pressure (Lin and Buehler, 2013) and have been successfully produced in large quantities (Enyashin and Ivanovskii, 2011; Zhu et al., 2013). Furthermore, due to the recent development in radialene and annulene chemistry, some GFMs have been successfully synthesized unto large area multilayer substrate film in form of low dimensional nanostructures, indicating it can be adapted for various applications (Tahara et al., 2013).

Considering the aforementioned properties of GFMs coupled with their uniform conduction pore sizes and pristine pore edge, some authors have recently reported the application of GFMs in water desalination and water purification (Kou et al., 2014; Mehrdad and Moosavi, 2019). However, the number of available reviews focusing on graphyne and its family members for water purification/desalination are still very scanty. It is therefore necessary to look into this special area of interest and review recent literatures reports in order to be able to identify knowledge gaps and research barriers for further improvement of both experimental and theoretical approaches currently being engaged. In this review, the recent advances in the application of GFMs-based membranes for water desalination are summarized and discussed focusing on the time frame from January 2013 till October 2022. Firstly, we discussed the vast and unique properties of GFMs to justify their suitability for desalination technologies. Thereafter, we outlined the common approaches and processes utilized in the investigation of graphyne-based membrane performance used for water desalination as established in literatures. Their outcomes in terms of water permeability and salt rejection were also considered. Lastly, we concluded with sections on current challenges, outlooks and opportunities associated with the continuous and sustainable development of graphyne and its family-based membranes for practical and large-scale applications.

## 2 Properties of GFMs

The properties of GFMs were briefly described in the introduction section. Basically, GFMs, just as graphene have several unique properties. These properties can be broadly classified into electronics, magnetic, mechanical and atomic, thermal, optical, and structural. The following sections briefly discuss these properties as they relate to various studies.

### 2.1 Electronics

One of the fundamental properties of materials is the electronic property. To achieve efficient performance in application, it is important to have proper understanding of the material electronic structure in question. Therefore, proper understanding of GFMs electronic structure is key to its performance. Theoretically, GFMs possess better and excellent electronic properties with a natural band gap around 0.44–2.23 eV compare to graphene with band gaps of zero (Malko et al., 2012; Sheng et al., 2019; Sani et al., 2020). The special presence of triple carbon-carbon bond in GFMs allows for reversed chirality properties and momentum shift of their Dirac cones making it possible to achieve tunability of their energy gap (Kim, 2012). These Dirac cones were uniquely found in the electronic structure of graphene, but are now demonstrated with first principles calculations to be present in GFMs (Malko et al., 2012). GFMs show both semiconductor and metallic behavior along zigzag and armchair directions, respectively. One key parameter that is important in the electronic properties of semiconductor materials is electron mobility and GFMs have shown intrinsic electron mobility (Gao et al., 2019; Li et al., 2020b). When different layers of GFMs are stacked using varying methods, they can influence their electronic properties. León and Pacheco (2015) studied the electronic properties of bilayers GFM with different stacking configurations of either its metallic or semiconductor form, it was observed that the band gap in the semiconductor form can be modulated. Zheng et al. (2012) examined bandgap and mobility (two related electric properties parameters) and different stacking behaviors of GFMs. They discovered that benzene ring stacked in the Bernal mode resulted in the most stable stacking mode for bilayer and trilayer GFMs. Their bandgaps are smaller than the intrinsic bandgap of the single-layer GFMs and when the stacking modes of the bilayer and trilayer were revealed, they displayed metallic characteristics. Other detailed studies featuring a natural band gap and superior electric properties of GFMs with different layers and stacking models has been reported (Matsuoka et al., 2017; Gao et al., 2018; Huang et al., 2018).

### 2.2 Mechanical

The prediction of GFMs structures have attracted many researchers in finding out more about their mechanical properties (Peng et al., 2014). Mostly responsible for their mechanical behavior is the presence of the acetylenic groups. These acetylenic linkages are largely influenced by the Young’s modulus and fracture stress percentages. Cranford and Buehler (2011) investigated the mechanical strength using molecular dynamics (MD) simulations with ReaxFF potential. Appreciable tensile strength and fracture strain were obtained for both arm-chair and zigzag loads. In another study (Yang and Xu, 2012), discovered the disparate impacts of the addition of acetylene groups of graphyne family on the mechanical performance through acting tensile loads on the architectures. For MD simulations in the armchair direction as opposed to zigzag direction, the fracture strain in the armchair loading case remains nearly unchanged whereas the tensile strength gradually reduces with longer acetylenic chains. On the other hand, the tensile strength remains almost the same under the zigzag loading condition, whereas the fracture strain increases minimally due to the presence of fewer bond linkages and low atom density in the molecular plane of GFMs. Ajori et al. (2013) reported the influences of vacancy defects on graphyne mechanical parameters such as strain, stress, Young’s modulus and Poisson’s ratio by employing MD simulations on the basis of Tersoff–Brenner potential function. Their results demonstrated that graphyne has reduced strength and stiffness compared to graphene. Wu et al. (2017b) applied MD simulations on the basis of AIREBO (Adaptive Intermolecular Reactive Empirical Bond Order) potential function to determine fracture stress, fracture strain, and stiffness with due consideration given to both armchair and zigzag load directions. It was revealed that the number of acetylenic chains between the hexagonal rings had great impact on the mechanical properties of GFMs. However, some studies have shown that the mechanical properties of GFMs can be temperature-dependent (Shao et al., 2012; Zhang et al., 2014), although this aspect of research is still ongoing. In-plane stiffness and Poisson’s ratio are also two important elastic parameters in discussing the mechanical properties of GFMs. When compared with graphene, GFMs usually possess smaller in-plane stiffness (Kang et al., 2011; Peng et al., 2012) while the Poisson’s ratios for the GFMs could range from 0.39 to 0.87 and are all larger than that of graphene (Wen et al., 2019). In all the studies considered, the mechanical parameters have proven that GFMs are mechanically stable, which qualifies them as suitable candidates for membrane materials and applications.

### 2.3 Thermal/thermoelectric

The thermal or heat transport property of materials applied in nanotechnologies and other related application is of utmost importance. Varying theories have been used to explain the thermal conductivity of GFMs such as non-equilibrium MD (Galamba et al., 2007; Font et al., 2021), Green−Kubo formula with equilibrium MD (Manjunatha et al., 2021), Boltzmann transport equation (Tan et al., 2015; Jiang et al., 2017) and so on. Zhang et al. (2012) studied the thermal conductivities (TC) of four GFMs and found that the presence of the acetylenic linkages in the GFMs caused excess reduction in their thermal conductivities due to the associated low atom density in the structures and weak single bonds. 6, 6, 12-graphyne out of the four graphyne-n sheets exhibited anisotropy in the TC, different from other graphyne-n sheets considered. Ouyang et al. (2012) investigated the thermal transport property of *γ*-graphyne nanoribbons and showed that the thermal conductance of *γ*-graphyne nanoribbons is approximately 40% lower than that of graphene and the conductance was insensitive to the acetylenic linkages. Hu et al. (2015) in their study on thermal conductivity of graphyne nanotubes revealed that the perfect *γ*-graphyne nanotube exhibits an unprecedentedly low thermal conductivity that is much lower than those reported for ordinary, defected, and chemically functionalized carbon nanotubes. More recently, Zhang. et al. (2017a) in their study on thermal conductivity of *δ*-graphyne showed that as the temperature increased, the thermal conductivity of δ-graphyne monotonically decreased because of the presence of the acetylenic linkages. The outcome from all these studies is identical as they all revealed the relative difficulty of heat transport in GFMs, which is largely associated with their low thermal conductivity. Another interesting related concept is the thermoelectric performance of GFMs. As established by past findings, GFMs possess smaller thermal conductivity than graphene. However, the former predictively showed good thermoelectric performance with high figure of merit because of the band gap in GFMs, which can significantly increase its Seebeck coefficient. Some of these findings are reported in literatures (Sun et al., 2015; Tan et al., 2015; Jiang et al., 2017). Generally, an ideal model for thermoelectric material should have a dimensionless figure of merit, ZT >3. A ZT value of 3 and 4.8 was obtained for p-type holes and n-type electrons in GFMs at 293K *via* combining MD and first-principles simulations together with Boltzmann theory, which indicates that GFMs are potential materials for high-powered thermoelectric devices (Sun et al., 2015).

### 2.4 Structural and stability

Essential to producing high-performance carbon-based nanoscale materials is the clear understanding of their structural and stability properties. Design optimization and variation of their structure such as edge functionalization and ribbon width can help enhance their structural and stability properties. In addition to this, their mechanical, chemical and electronic properties are also improved in the process (Li et al., 2014). Bai et al. (2011) investigated the stability and structural properties of graphdiyne nanoribbons using density functional theory (DFT). Based on the calculated cohesive energies, one-dimensional graphdiyne nanoribbons displayed more stability than other two-dimensional graphdiyne slab in view of energy. Also, graphdiyne nanoribbons with zigzag edges are more stable than those with the armchair structures. Furthermore, the stability and structural properties of a set of fluorographynes (a typical GFM) were investigated by Enyashin and Ivanovskii (2013). By employing DFT band structure calculations, their results revealed the effect of fluorine on graphyne sheets. The stability of the fluorographynes increases as the ratio of fluorine-carbon becomes higher. Meanwhile, increasing the sp^1^ atom in the graphyne sheet created some reduction effect in the stability of both the pristine graphyne sheet and the fluorinated derivatives. Solis et al. (2019) examined the structural stability of graphyne and graphdiyne nanoscrolls through MD simulations. They observed that stable nanoscrolls could be created for all the studied structures resulting from the higher structural porosity of the graphyne/graphdiyne compare to graphene, and as a consequence, the π−π stacking interactions decrease. In another study, a relationship was established between the elastic property of the GFM and its structure. As the percentage of acetylenic linkages increases, the in-plane stiffness and layer modulus decrease, while the Poisson’s ratio increases. Also, dependence of structure was attributed to bond density change (Qu et al., 2017).

### 2.5 Magnetic

The magnetic properties of carbon materials have been considerably studied because of their applications as light non-metallic magnets and promising potential in spintronics (Chen et al., 2017; Gao et al., 2019). Generally, the zigzag direction loading graphyne nanoribbons usually bear a magnetic semiconductor ground state with ferromagnetic order both at the edge and the opposite spin directions, while the armchair direction loading graphyne nanoribbons are non-magnetic semiconductors with its band gap being a function of its width (Li et al., 2020b). The electronic structure of GFMs is well regulated by the adsorption of 3d transition metal atoms and in the process, an excellent magnetic property is impacted *via* spin polarized semiconductors.

Zhang et al. (2017b) investigate practically the paramagnetic characteristic of a pristine GFM material and GFM material doped with nitrogen. The results revealed that the paramagnetic characteristic of the GFM material doped with Nitrogen was increased, and show a clearer saturated magnetic moment value than the pure GFM material. However, when an asymmetric pyridinic nitrogen was introduced *via* substitution reaction, a huge local magnetic moment (0.98 μB) was obtained. This suggests that nitrogen of pyridine type is more favorable to improve magnetism of GFMs than just doping N sites. In a subsequent study, the authors obtained a hybrid composite system based on GFM materials and ferrous ions (Fe) doped through a simple and affordable synthetic route showing forth a favorable ferromagnetic characteristic (Zhang et al., 2017b). Despite some achievements made in the explorative studies of the magnetic properties of GFMs, more prospect can be achieved for excellent exhibition of magnetic properties applicable in spintronic devices.

### 2.6 Water purification and desalination

One of the interesting properties of GFMs besides all the aforementioned qualities is their ability to serve as outstanding separation materials in water purification and desalination. The ability to adjust their pore structures for easy permeation of water molecules makes them great prospect in water desalination and purification. In addition, they have high tunable surface energy and are super hydrophobic (Huang et al., 2018). Compare to graphynes, graphdiynes are semipermeable with several continuous channels that can allow the passage of some reasonable quantity of water at certain applied pressures while effectively rejecting ions (Zhu et al., 2013; Bartolomei et al., 2014). These nanopores channels can be improved *via* negative charging or functionalization to enhance their water permeability and salt rejection (Azamat et al., 2020). One of the key performances of the special triple carbon-carbon bond present in GFMs is the allowance for the momentum shift of their Dirac cones which makes it possible to achieve tunability in their energy gap; a condition that favors electron mobility hence, their surface functionalization (Kim, 2012; Kang et al., 2019). Some works have highlighted the attractiveness of GFMs as molecular filters for water purification and desalination. These works are elaborately presented in Section 4.

## 3 GFMs-based membranes: Computational methods

Reports on analysis and performance of graphyne-based membranes have largely been achieved *via* theoretical and computational approaches. These approaches are usually helpful when the objective is prediction or there is need for understanding an unclear phenomenon, or to explain an astonishing experimental observation (Cohen-Tanugi and Grossman, 2015). Computational approaches are also helpful as guidelines in indicating the best region or area of a material phase in a given application and in making decision as regarding the selective choice of an experimental material especially in the case of graphene sheet. Many prediction studies on graphyne and its family members have been reported. Here we focus on the most common computational modeling tools used in water desalination using membrane technology. The most common computational modeling tools used include molecular dynamics (MD) simulations, quantum mechanics (QM) calculations and combination of QM/MD simulations. DFT computations is the most used quantum mechanics method. In some other applications and subjects of concern, analytical models or finite element approaches can be employed as computational tools.

### 3.1 Molecular dynamics (MD) simulation

Molecular dynamics (MD) simulation (also known as molecular mechanics, MM) is a significant computational approach that enables the study of particle interactions between atoms by solving Newton’s equations of motion within a given force field. They are useful in examining the implication of the intermolecular interactions (bond stretching and angle bending) and intramolecular interactions (van der Waals and electrostatic interactions) on the behavior of ions and molecules in a system (Hosseini et al., 2019). In addition, the behavior of relatively large molecular systems even up to 10^9^ atoms can be investigated within a physical reasonable timescale of 1 ns and 1 μs. MD simulations works well with several inputs categorized under three factors: i) Initial configuration of the system, ii) set of forcefields and iii) sets of constraints. The positions, atomic elements, bonding states and partial charges of all atoms constitutes the initial configuration of the system while the constraints include the geometric boundary conditions, a thermodynamic ensemble, and a thermostat or barostat (Cohen-Tanugi and Grossman, 2015). MD simulations in recent decade has begun to gain profound attention in water desalination application for nanomaterials such as high-aligned and high-density CNTs, zeolites, boron nitride and molybdenum disulfide (MoS_2_) (Li et al., 2008; Zhang et al., 2018; Oviroh et al., 2021). In a recent explanation of Cohen-Tanugi and Grossman (2015) on MD simulation study of nanoporous graphene used as an RO membrane, two water reservoirs was divided with a nanoporous graphene layer and a driving force acting as pressure or electric field, was applied to the water and ions. The trajectories provided profound information on the water flux and the solute, which were vital to the membrane water permeability and salt rejection estimation. Insights were also given on the physical dynamics of the system such as the fluid properties, membrane behavior and the physical mechanism for salt permeation.

### 3.2 Quantum mechanics (QM) calculation/simulation

QM simulations are best and appropriate techniques for structures wherein bonding, chemical reactions or electronic properties are of matter of concerns. The explicit nature of atoms (nuclei and electrons) in QM aids the properties of materials computation to be done with accurate quantum effects. As stated earlier, DFT is the most common quantum mechanics method, and it is an efficient tool in evaluating the interactions between molecules with active consideration of quantum effects. For predicting the ground-state electron density of atomic systems, DFT uses functionals to approximate electron correlations and exchange energies (Cohen-Tanugi and Grossman, 2015). DFT has been widely employed in graphene related studies both within and outside the scope of water desalination (Compagnini et al., 2011; Patra et al., 2016). However, its application in water desalination through graphyne-based membrane is still ongoing.

One major demerit of the QM methods is their expensive computational cost, which mostly results in the consideration of only small systems containing hundreds of atoms. However, compared to MD simulations study in water desalination and gas separation applications, they are effective in overcoming the energy barrier faced by individual molecules permeating through graphyne pores during system operation (Qiu et al., 2019). Other advance QMs, which provide higher accuracy in simulation with extended increased computational time are Quantum Monte Carlo and GW calculations (Hedin and Lundqvist, 1970; Grossman, 2002; Wang, 2011; Golze et al., 2019).

### 3.3 Hybrid quantum mechanics/molecular dynamics simulation

In an attempt to overcome the individual limitations of MD and QM simulations, the concept of combining these two useful computational tools have emerged. They were first proposed and introduced by Warshel and Levitt as a multiscale computational tool, which allow a reliable QM calculation on a system with a realistic modeling of the complex environment (Warshel and Levitt, 1976) and named it combined quantum mechanical and molecular mechanical (QM/MM) method or hybrid quantum mechanical and molecular dynamics QM/MD methods. The MD simulations which from tens of picoseconds to hundreds of nanoseconds are needed to gain converged statistical sampling for free energy calculations, alongside the change in the electronic structures of the system (in terms of bond forming or breaking processes) requires quantum mechanics tool such as DFT. This therefore confines the application of MD to a small number of atoms for a short time (Shen and Yang, 2018). Similarly, excessive computational cost is incurred with the QM method (Qiu et al., 2019) thus justifying this innovative concept of hybrid QM/MD.

**FIGURE 3:**
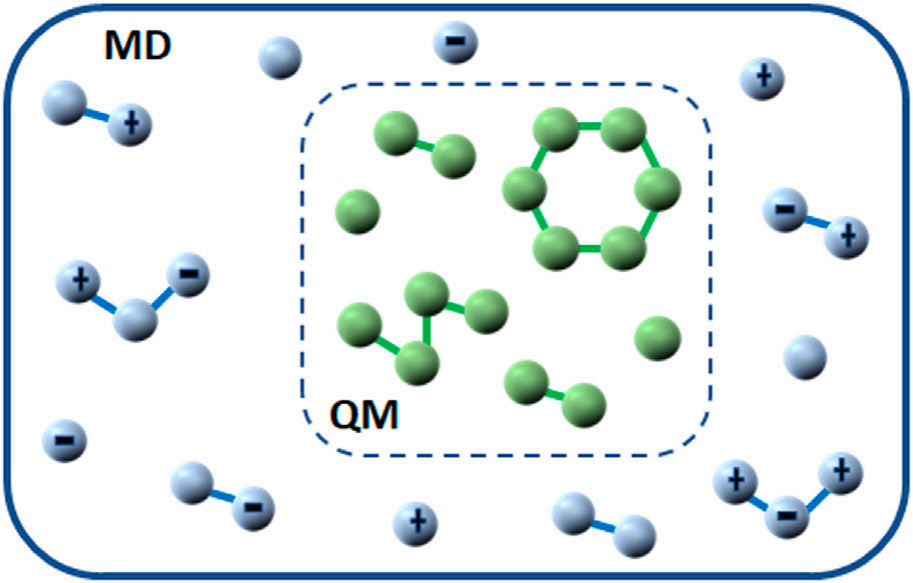
Hybrid QM/MD strategy.

**FIGURE 4:**
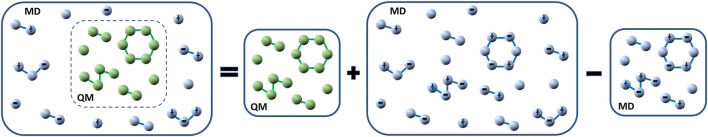
Subtractive QM/MD coupling mechanism.

Figure 3 illustrates the hybrid QM/MD strategy as originally introduced by Warshel and Levitt (1976) in which a system, divided into sections and explained at various levels of theory, is considered the local character of most chemical reactions in condensed phases. Therefore, it is easy to make a distinction between a “reaction region” having atoms that are directly and actively involved in the reaction and a “spectator region” where the atoms do not directly participate in the reaction (Groenhof, 2013). Furthermore, three types of interactions occur in hybrid QM/MD potential energy system as depicted in Figure 4. The magenta shaded portion represents the interactions between atoms in the QM region, the light green shaded portion represents the interactions between atoms in the MM region and the orange shaded portion represents the interactions between QM and MD atoms. Individually, the interactions between atoms within the QM and MD regions are unambiguous to explain while those of the two subsystems are relatively complex to describe (Groenhof, 2013). Notwithstanding, a careful look at Figure 3, relatively explains the complexity, such that the QM/MM energy of the total system is assumed to be equal to the energy of the isolated QM subsystem (evaluated at the QM level), in addition to the energy of the complete system (evaluated at the MM level), minus the energy of the isolated QM subsystem (evaluated at the MM level).

In recent times, QM/MD methods, which was firstly conceived in the environment of biomolecular simulation studies (Friesner and Guallar, 2005; Hu and Yang, 2009; Cui, 2016; Lu et al., 2016) has been extended into other areas of solid-state chemistry, solution chemistry, and material science (Gao, 1996; Harschend et al., 2004; Keal et al., 2011; Hofer et al., 2012; Golze et al., 2015; Hofer and Tirler, 2015). Specifically, QM/MD methods has the capacity to model time-dependent behaviors in situation with limited empirical potential functions. However, Newton’s equations of motion is used to determine the movement of each atom while DFT and *ab initio* (types of QM methods) are used to calculate the interatomic forces (Qiu et al., 2019).

## 4 GFM-based membranes: Application in water desalination and purification

One of the main areas of GFMs application, which is currently receiving much attention, is in water purification and desalination. For over two decades, advances have been made in the utilization of graphene for several applications of science and engineering, and most especially in water filtration and desalination because of its attractive and advantageous properties compared to some other nanomaterials (Aghigh et al., 2015). However, some steps have been employed in recent years to improve their performances in membrane desalination application. One of such bold steps is the utilization of graphene derivatives, and this is anchored on the minimum possible membrane thickness that can be achieved with GFM, which in turn tend to favor high permeance with appreciable salt rejection (Qiu et al., 2019). This section thus, systematically summarizes the recent research carried out from 2013 till present using GFMs for water purification and desalination from theoretical and computational viewpoints (Table 1). Some essential information on the experimental preparation and simulation process adopted for graphyne-based separation membranes were highlighted. However, emphasis was placed on two most important parameters used to characterize the performance of membrane in desalination process, which are permeability and/or selectivity.

**TABLE 1:** Summary of reported studies on GFMs membrane material applied in water purification and desalination.

GFM membrane material type	Molecular medium	Rejected ions/salts	Permeability	Rejection (%)	Pressure applied (MPa)	Computational methods	Ref
α−/β− Graphyne^a^, Graphidyne^a^, Graphyne-3/-4	Water	Na^+^, Cl^−^, Mg^2+^, K^+^, Ca^2+^	∼10 L/cm^−2^/day/MPa	100% rejection for graphyne-3 and a little lower for graphyne-4	0–250	MD	Xue et al. (2013)
Graphyne-3/-4/-5	Water	Na^+^, Cl^−^	NS	NS	0–600	MD	Kou et al. (2013)
Graphyne-3/-4/-5/-6	Water	NaCl, CuSO_4_, C_6_H_6_, CCl_4_	2.5–4.5 × 10^−9^ m.Pa^−1^.s^−1^	75% above rejection for all the graphyne-n tested	50	MD	Lin, and Buehler, (2013)
Graphyne-3/-4/-5/-6	Water	Na^+^, Cl^−^	13 L/cm^−2^/day/MPa	100% rejection for graphyne-4		MD, QM/MD	Zhu et al. (2013)
	Water	Na^+^, Cl^−^	35–130 L/cm^−2^/day/MPa for graphyne-3/-4/-5	100% rejection for graphyne-3 and a little lower for graphyne-4	0–350	MD	Kou et al. (2014)
Graphyne-3/-4/-5/-6	Water	Na^+^, Cl^−^	39.15 L.cm^−2^ h^−1^	100% rejection for graphyne-3 and lower for other graphyne-n membranes tested	NS	MD	Zhang and Gai, (2015)
Graphyne-3/-4/-5	Ethanol	H_2_O	NS	NS	NS	MD	Liu et al. (2016)
Pristine/charged graphyne-3/-4/-5	Water	Na^+^, Cl^−^	13.5 L/cm^−2^/day/MPa	100% rejection for graphyne-3 and lower for other graphyne-n membranes tested	0–150	MD	Wu et al. (2017a)
Graphyne-3/-4/-5	Ethanol	H_2_O	NS	NS	100	MD, QM	Yang et al., 2017
Bilayer graphyne-3/-4	Water	Na^+^, Cl^−^	∼60 L/cm^−2^/day/MPa	100% rejection for graphyne-3 and a little lower for graphyne-4	50–200	MD	Akhavan et al. (2018)
Bare and hydrogenated α-graphyne and graphyne-2/-3/-4	Water	Na^+^, Cl^−^	85 L/cm^−2^/day/MPa	>90% rejection for all tested membrane except graphyne-4	0–1,000	MD	Raju et al. (2018)
Graphyne-3/-4/-5	Ethanol	H_2_O	NS	NS	40–100	MD	Zhang et al. (2018)
Bare and functionalized graphyne-3/-4/-5	Water	Na^+^, Cl^−^	17 L/cm^−2^/day/MPa	100% rejection for F-functionalized graphyne-4 and a little less for bare graphyne-4 and others tested	100–200	MD	Mehrdad and Moosavi, (2019)
Functionalized *?*-graphyne-1	Water	Na^+^, Cl^−^	8953 L/m^−2 ^h bar	100% rejection for all the functionalized γ-graphyne-1 membrane tested at < 7.5 MPa	0–50	MD	Azamat et al. (2020)
Pristine graphdiyne	Water	Na^+^, Cl^−^	565.37 L/m^−2 ^h bar	99.41	400	MD	Baghbani et al. (2020)
Anisotropically nanoporous graphyne and graphyne-3/-4/-5	Water	Na^+^, Cl^−^	7.98–47.14 L/cm^2^/day/MPa	100% rejection for graphyne-3 and ANGM-n (*n* = 1, 2, 3) membranes and others except graphyne-5, could reject >98.2% of ions up to 150 MPa	50–250	MD	Nematipour et al. (2021)
Functionalized graphenylene	Water	Na^+^, Cl^−^	11,032 L/m^−2^ h bar	99.4% rejection at 10 MPa for fluorinated functionalized pore	0–100	MD	Jahangirzadeh et al. (2022)

^a^ These are graphynes reported as unsuitable for separation and NS means “Not Supplied”.

### 4.1 Pristine graphyne-n membrane water desalination

**FIGURE 5:**
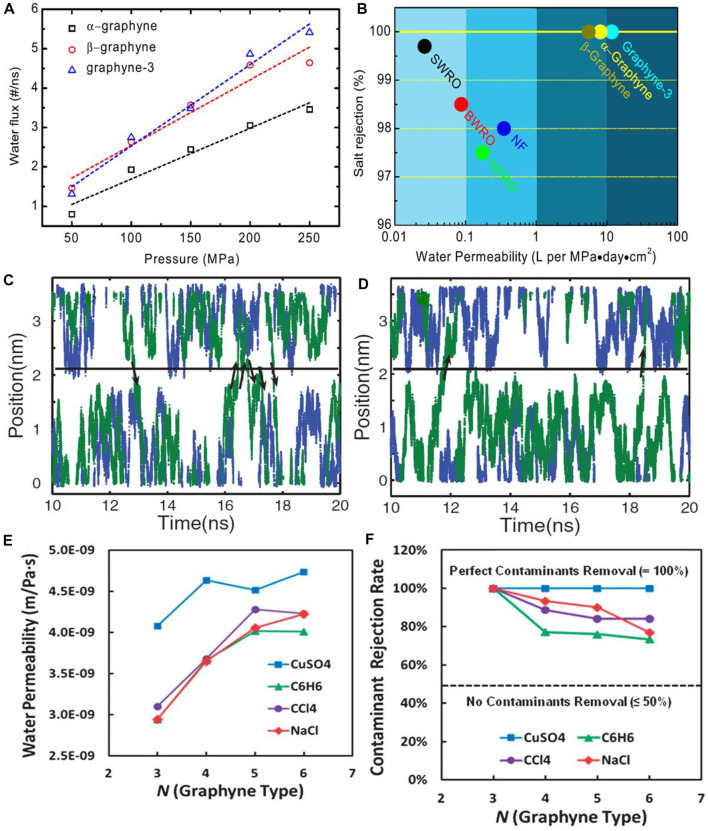
**(A)** Water permeability across α-graphyne, *β*-graphyne, and graphyne-3 as a function of external hydrostatic pressure. **(B)** Performance comparison between α-graphyne, *β*-graphyne, and graphyne-3 and some conventional RO desalination membranes **(C)** water molecule motion trajectories without hydrostatic pressure for graphyne-2 (blue points) and graphyne-3 (green points) **(D)** water molecule motion trajectories with hydrostatic pressure for graphyne-2 (blue points) and graphyne-3 (green points). The black arrows show the direction of water molecule through membrane **(E)** water permeability for various graphyne-n membranes (*N* = 3–6) during the contaminants filtration simulation **(F)** contaminant rejection rates through various graphyne membranes under Δ*P* = 50 MPa (Figures **(A,B)** were adapted from Xue et al., 2013; **(C,D)** from Kou et al., 2013, and **(E,F)** from Lin and Buehler, 2013).

Graphyne is one-atom-thick carbon allotrope that resemble graphene and it has been engaged in its bare/pristine form (i.e., unfunctionalized) in water desalination. Guo’s group in 2013 (Xue et al., 2013) reported the desalination performance of five various graphynes *via* MD simulations and these includes *a*-graphyne, *ß*-graphyne, and three analogues of γ-graphyne namely graphdiyne, graphyne-3, and graphyne-4. Prior to the experimental analysis, the geometric structures of the graphynes were optimized with DFTs and the constructed rectangular graphyne sheet was fixed at origin during MD simulations. The MD simulations were carried out and visualized by methods established in literatures (Humphrey et al., 1996; Phillips et al., 2005). Other parameters such as water modelling, carbon atoms particles and simulations testing with sp^2^ carbon in the AMBER force field were established from previous reports (Jorgensen et al., 1983; Pearlman et al., 1995; MacKerell et al., 1998). The water permeability was firstly measured across the five graphynes with ranging pressures from 0–250 MPa, and included in the simulation system is Na^+^ and Cl^−^ with salt concentration of 1.2 M. The water fluxes measure as a function of the external hydrostatic pressure exhibited a linear relationship with the pressure as depicted in Figure 5A. As revealed in the work, *ß*-graphyne can transport water about 31% faster than α-graphyne because of its larger effective internal pore area obtained by assuming carbon atom van der Waals radius of 1.7 Å. Meanwhile, graphdiyne was impermeable to water or ions even at pressure of 250 MPa, which connotes that it is a poor candidate for desalination. However, graphyne-3 and graphyne-4 exhibited higher water permeability compared to *α*-graphyne and *ß*-graphyne in the study. With respect to salt rejections, *α*-graphyne, *ß*-graphyne, and graphyne-3 were reported to reject 100% Na^+^ and Cl^−^ salt ions. Likewise, at increased pressure (up to 250 Mpa) and salt concentration (from 1.2–3.6 M), their ion rejection ability was still intact thus, indicating their independence of salt concentrations and operating pressures. This observation is in contrast to the behavior of CNTs at a concentration higher than 0.01 M (Fornasiero et al., 2008). Moreover, their rejection of other salt ions present in sea water was exceptional, as none of the ions could permeate through them. Graphyne-4 on the other hand can be permeated by water rapidly than other graphynes, but at a compromised salt rejection. A performance comparison of α-graphyne, *ß*-graphyne, and graphyne-3 with conventional RO membranes (seawater RO, brackish water RO, high-flux RO and NF) revealed that the permeability of these graphynes at 100% salt rejection can provide two orders of magnitude higher than that of commercial RO membranes as shown in Figure 5B. Therefore, the monolayer graphyne-3 membrane having the best performance was validated to be a promising candidate for application as membranes for water filtration and desalination (Xue et al., 2013).

Further work on the spontaneous and continuous permeation of water molecules through a single-layer graphyne-3 membrane by molecular dynamics simulations was reported by Kou et al. (2013). Graphyne-3 with a dimension of 6.0 by 6.25 nm corresponding to its length and width, was immersed in a water bath. The monolayer graphyne was formed by replacement of carbon atoms and carbon-carbon interactions in the acetylenic linkages, which was described using AIREBO potential. Graphyne-2 and varying CNT diameters with length of 1.35 nm each were also considered for comparison. By using Gromacs 4.0.7 (Hess et al., 2008), the MD simulation was performed and the simulation system was filled with 4018 SPC/E model water molecules as described in Berendsen et al. (1987). Water molecule motion was tracked along the *z*-axis as a function of time in a simulation system hosting either graphyne-2 and -3 membranes with or without hydrostatic pressure. It was observed that the water molecule without the application of hydrostatic pressure navigate randomly and permeate the graphyne-3 membrane system after sometime, which was not the case in graphyne-2 system (Figure 5C). Meanwhile, after applying hydrostatic pressure, water molecule navigates readily along *z-*axis and permeate the membrane easily in graphyne-3 system. However, for graphyne-2 system, the water molecules could not permeate the membrane within the simulation period (Figure 5D). Furthermore, the net fluxes of water through graphyne-2, graphyne-3 and the CNTs membranes [(5, 5) CNT, (9, 0) CNT, and (10, 0) CNT] were closely related to their permeabilities. The result was in the order: (10, 0) CNT > (9, 0) CNT > graphyne-3 > (5, 5) CNT >graphyne-2 with the net water flux of graphyne-2 being zero (which is in tandem with the observation made in Figure 5). There was easy movement of water molecules *via* the monolayer graphyne-3 membrane than the (5, 5) CNT membrane. The net water flux in graphyne-3 membrane is 27.5 ns^−1^, but 13.5 ns^−1^ in (5, 5) CNT, even though they have similar nanopore diameters. Although, the net water fluxes of (10, 0) and (9, 0) CNTs were higher than that of graphyne-3 membrane, their work showed that single layer graphyne-3 membrane displayed a higher water permeability than CNT membranes having the same nanopore diameter (Kou et al., 2013).

The study conducted by Lin and Buehler (2013) took a different approach as 2D nanoweb-like graphyne membrane was used for water purification and also to remove contaminants from wastewater and seawater *via* MD simulations. They examined the relationship between the mechanical ability, filtration mechanism and rejection performance using monolayer graphyne membranes with various acetylenic linkage lengths (*N* = 3–6). Large-scale Atomic/Molecular Massively Parallel Simulator (LAMMPS) software package was used for the biaxial mechanical tensile tests while the atomistic simulations were done using ReaxFF potential developed by Chenoweth et al. (2008) to model carbon–carbon and carbon–hydrogen interactions. Gromacs 4.0 software package was used for water desalination process simulations (Hess et al., 2008). The representative contaminants examined include .6 M each of copper (II) sulfate (CuSO_4_), benzene (C_6_H_6_), and carbon tetrachloride (CCl_4_) while NaCl (completely ionized into Na^+^ and Cl^−^) was used as the saline water. The biaxial tensile tests of the graphyne membranes exhibited superior mechanical strength with ultimate stress of 16.7–32.3 GPa, which proves their high tolerance to deformations from the membrane installation process. More importantly, the water permeability of the graphyne membranes ranges from 2.9–4.5 × 10^−9^ mPa^−1^ s^−1^, which increases as the acetylenic linkage number increases. However, this flow rate started declining after reaching 5 and 6 acetylenic linkage as shown in Figure 5E. Also, the flow across the graphtriyne membrane exhibited an optimal purification performance which ranges from 3.0–4.0 × 10^−9^ mPa^−1^ s^−1^ and could still reject contaminants excellently under the applied hydrostatic pressures. Furthermore, the graphyne membranes showed higher rejection for CuSO_4_ and NaCl (which are hydrophilic in nature) compared to C_6_H_6_ and CCl_4_ (which are hydrophobic in nature), which was linked to their different interaction strengths with water molecules. The rejection rate for all contaminants decreases as acetylenic linkage number increases, thereby following the trend CuSO_4_ > NaCl > CCl_4_ > C_6_H_6_ as shown in Figure 5F.

The work of Zhu et al. (2013) corroborates the work reported by Xue et al., 2013, *via* the use of extensive MD simulations to examined the performance of *γ*-graphyne for water desalination at high rate. Parameters used for the graphyne-n sheet with acetylenic linkage number of 1–6 in their study were adapted from Narita and Nagai (1998). SPC/E model described in Berendsen et al. (1987) was used for water in the simulation system. Interaction parameter for graphyne used for Na^+^ and Cl^−^was adapted from the Amber99 force field while the non-bonding interactions are modeled by the Lennard-Jones (LJ) and Coulomb potentials. Overall system simulation was achieved using Gromacs 4.0 software. As observed by the authors, no water permeates through graphyne-1 or graphyne-2 membrane because of their small nanopores but at graphyne n with n ≥ 3, a breakthrough in water permeation was achieved. Water flow rate was also measured, and it increases linearly with pressure as low as around 5 MPa. Further analysis was carried out by examining the single-pore permeability, which was defined as the flow rate per unit pressure per nanopore. A stepwise increase was observed as the pore size increases from *n* ≥ 3, which was described as a special feature of ‘‘quantized’’ water flow through the graphyne membranes. Water flow per area across graphyne-4 is greater than graphyne-5 and graphyne-6 because of their higher distribution density of nanopores, having a maximum value of 13 L/cm^2^/day/MPa, which is three orders of magnitude higher than commercial RO and 10 times higher than nanoporous graphene (Pendergast and Hoek, 2011). Moreover, all the graphyne membranes exhibited high salt rejection excluding graphyne-1 or graphyne-2, which were not tested. Graphyne-3 and graphyne-4 show a 100% rejection of Na^+^ and Cl^−^ salt ions, which was partly in agreement with the findings of Xue et al. (2013), while there were slight reductions in the salt rejection of graphyne-5 and graphyne-6 membranes. In addition, salt rejection efficiency was observed to slightly increase with applied external pressure due to the high computed passing ion energy barrier transported *via* the graphyne membrane nanopores. Their study concluded that the “quantized” nature of water flux through the membranes was responsible for their excellent performance (especially in graphyne-4).

Sequel to the findings on the assessment of water permeation through the two graphyne membranes (graphyne-2 and graphyne-3) and CNTs membranes (Kou et al., 2013), Kou et al. (2014) extend their study to examine the potential of water desalination (both water permeability and NaCl salt rejection) *via* three graphyne-n membranes (*n* = 3, 4, and 5). The MD simulations was done using Gromacs 4.0.7 as previously established (Hess et al., 2008). As observed during the simulated period, water containing Na^+^ ions could permeate the three membranes at different rate and with different fresh water production capacity. Graphyne-3 among others yielded only fresh water without Na^+^. In addition, the salt rejection capacity tested under hydrostatic pressure of 0–350 MPa showed that graphyne-3 membrane had a perfect 100% salt rejection while graphyne-4 and graphyne-5 membranes slightly varied. This outcome contradicts the results reported by Zhu et al. (2013) who claimed that graphyne-4 membrane could also yield 100% NaCl rejection. Though, further investigation by Kou et al. (2014) showed that graphyne-4 could also yield 100% salt rejection. However, Qiu et al. (2019) in their review attributed this inconsistency to different force field parameters used during MD simulations system set-up.

As a continuum to the studies of Lin and Buehler (2013), Kou et al. (2013) and Kou et al. (2014), water and salt permeability of monolayer graphynes-n (*n* ≥ 3) was investigated by Wu et al. (2017a) using MD simulations. MD simulations system was set up using established procedures documented in previous studies (Kou et al., 2013; 2014; Lin and Buehler, 2013; Xue et al., 2013). Desalination was carried out at high salt concentration and varying hydrostatic pressure. It was reported that water transport across graphyne-4 and graphyne-5 membranes are more than through graphyne-3 membrane during the simulation time of 4 ns. This increase in graphyne-4 and graphyne-5 membranes are associated to bigger van der Waals than in graphyne-3 membrane. The same observation was made for water flux per vdW pore quantitatively analyzed across the graphyne-n membranes. Furthermore, the augment of hydrostatic pressure resulted in increase in the water transport rates across all the monolayer graphene membranes, which was in tandem with previous study (Xue et al., 2013). On the other hand, graphyne-3 maintained salt rejection ratio of 100% at various hydrostatic pressure while there was notable decrease in those of graphyne-4 and graphyne-5 membranes (with graphyne-5 membrane showing higher degree of declination). This results support previous arguments indicating the suitability of graphyne-3 and graphyne-4 membranes for seawater desalination (Kou et al., 2013; 2014; Lin and Buehler, 2013).

According to Xue et al. (2013), graphdiyne was impermeable to water or ions even at pressure of 250 MPa, thus, making it an unsuitable candidate for desalination. However, Baghbani et al. (2020) further investigated the suitability of pristine graphdiyne membrane (without functional and chemical groups on its pore surface) for seawater desalination *via* MD simulation study. DFT was used to optimize the functionalized graphynes using the B3LYP theory level with the aid of GAMESS software (Schmidt et al., 1993) to obtain partial charges of all atoms. NAMD 2.18 software package was used for all the MD analyses (Phillips et al., 2005), while TIP3P model (Jorgensen et al., 1983) was used to model water interactions. Others parameters for ions and graphdiyne sheets were adapted from the CHARMM36 force field. Outcome of the experiment suggest that the graphdiyne membrane was completely impermeable at applied pressure less than 150 MPa, which is in agreement with the work by Xue et al. (2013) who reported no water passage up to 250 MPa. Interestingly at 150 MPa and even up to 650 MPa, Baghbani et al. (2020) showed that water flux could be observed, and continued to increase with increasing pressure gradient. In addition, the water permeability of the pristine graphdiyne nanosheet membrane reached 565.37 L/m^2^ h bar at 400 MPa, with corresponding salt rejection of 99.41%. At pressure less than 400 MPa, 100% salt rejection was realized and this performance is comparable to conventional RO system. Interestingly, this outcome exceed the performance of both graphyne-4 (90% salt rejection) and graphyne-5 (60% salt rejection) reported by Kou et al. (2014) These results thus reflects the potentials of graphdiyne nanosheet for application in seawater desalination.

**FIGURE 6:**
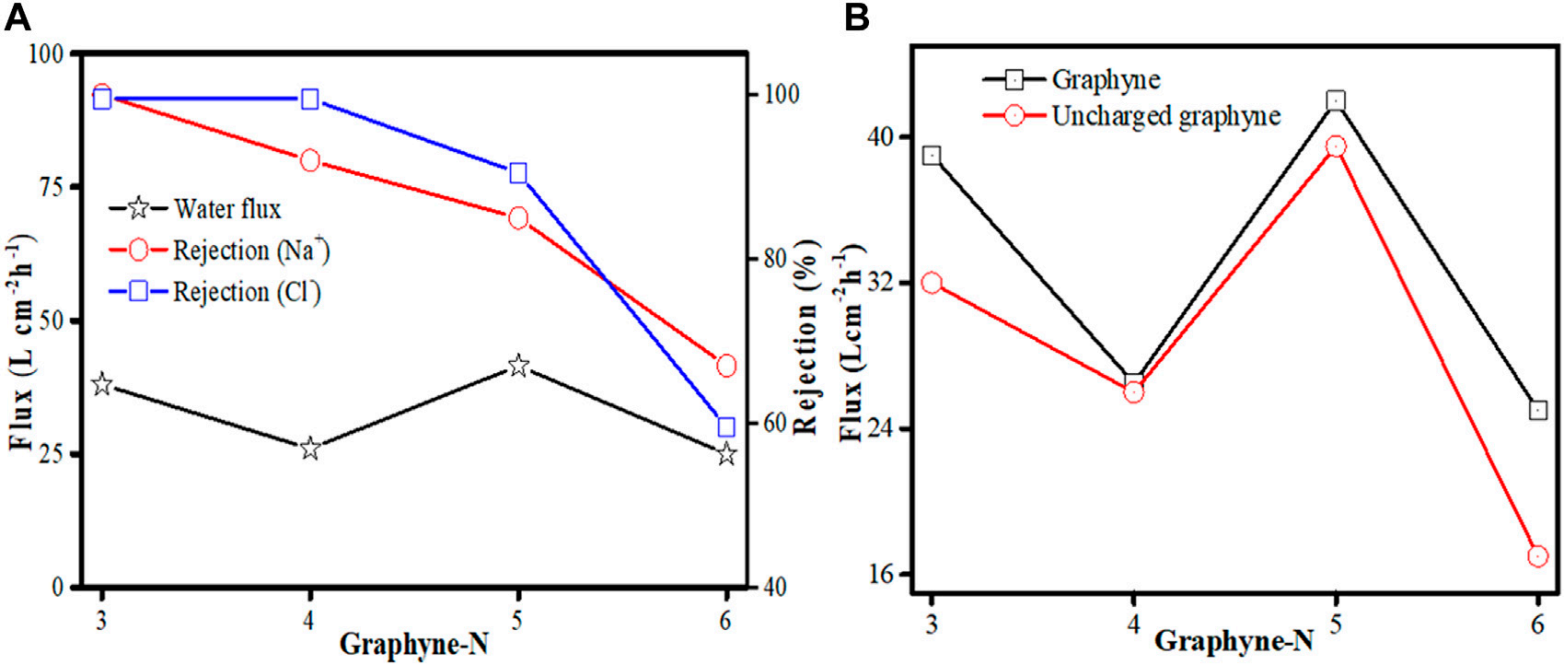
**(A)** Water flux and rejection of Na^+^ and Cl^−^ of graphyne-n (at *n* = 3, 4, 5, and 6). **(B)** Water fluxes of charged and uncharged graphyne-n membranes (at *n* = 3, 4, 5, and 6) (adapted from Zhang and Gai, 2015).

Advances was made on the application of graphyne membrane for investigating brine separation performance by Zhang and Gai (2015) in a forward osmosis (FO) system. MD simulations was used to obtain information on graphyne-n (where *n* = 3, 4, 5, and 6) membranes. Pure water and 5% NaCl were used as the feed and draw solutions, respectively. COMPASS force field and atom-based methods was applied in the calculation of the electrostatic and van der Waals effects. As observed from the simulated FO process, graphyne-3 membrane can yield up to an average water flux of 39.15 Lcm^−2^ h^−1^ with a rejection maintained at 100%. Similarly, graphyne-4, graphyne-5, and graphyne-6 membranes all have appreciable water fluxes slightly greater than or less than that of graphyne-3 but have lower salt rejections (Figure 6A). Considering the effect of charge property of each graphyne membrane on water transport, It was observed that the water fluxes of the charged membranes were higher than those of their uncharged counterparts (Figure 6B), thus making charged graphyne-n (*n* = 3, 4, 5, and 6) membranes advantageous for water transport in FO systems. Overall, their study shows that graphyne-3 possess excellent separation performance for brine separation while graphyne-n (*n* = 4, 5, and 6) are presumed to be good candidates for brine separation of lower salt rejection demand.

Studies on graphynes were not limited to only monolayer graphyne membranes as highlighted in the aforementioned works. The performance of bilayer graphyne membranes in water transport and desalination has been investigated. Akhavan et al. (2018) through a non-equilibrium molecular dynamics simulations investigated water desalination performance using double-layer graphyne membrane. Simulation parameter selection and set-up follows recorded procedures (Narita and Nagai, 1998; Cohen-Tanugi and Grossman, 2015). SPC/E model was used for water molecules and adapted OPLS parameters were applied for Na^+^ and Cl^−^ions (Berendsen et al., 1987; Lin and Buehler, 2013). All simulations were carried out using Gromacs package version 4.6.1 (Hess et al., 2008). The simulation results show that for both single and double-layer membranes, the water flow rate through the graphyne-4 membrane doubled that of graphyne-3 membrane, even though the addition of second layer membrane lessen the permeation by 50%, which is a factor of the layer spacing. At layer spacing of 0.35 nm, the flow rate reduced minimally while at spacing of 0.6 nm, the flow rate draws close to zero. For the salt rejection, graphyne-3 membranes displayed 100% rejection at all pressure ranges and layer spacing values while graphyne-4 membranes rejection is lower as expected with pressure and layer spacing grossly influencing its rejection performance. This agrees with other simulated desalination results (Lin and Buehler, 2013; Xue et al., 2013; Kou et al., 2014).

**FIGURE 7:**
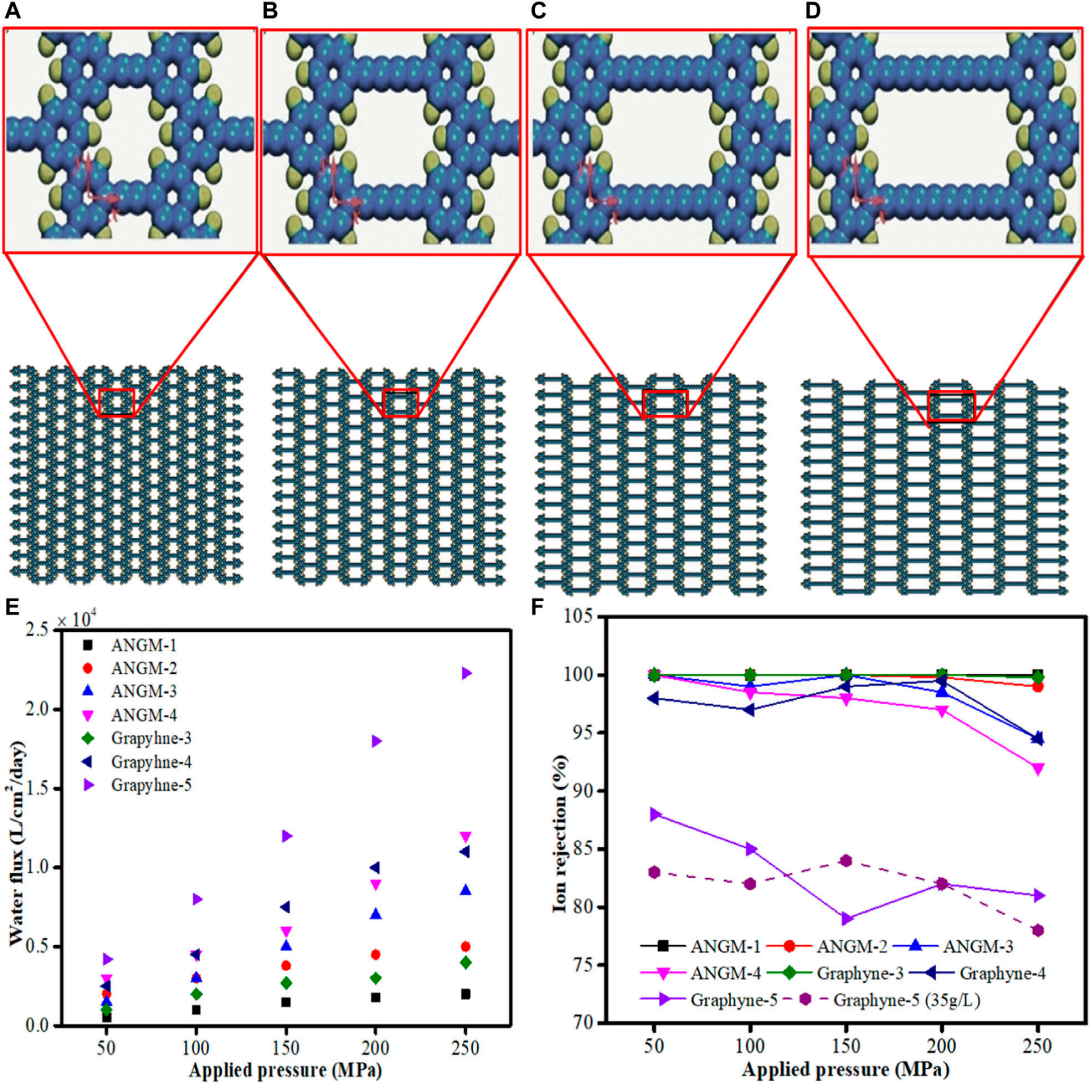
Schematic representation of **(A)** ANGM-1, **(B)** ANGM-2, **(C)** ANGM-3, and **(D)** ANGM-4 membranes. **(E)** Water flux of ANGM and graphyne membranes for various external pressures **(F)** Ion-rejection rate of ANGM and graphyne membranes as a function of external pressure (Adapted from Nematipour et al., 2021).

A novel concept of efficient water desalination using graphyne membranes was introduced by Nematipour et al. (2021). The authors investigated the feasibility of using anisotropically nanoporous graphyne membranes (ANGMs) to desalinate water *via* MD simulation process. These membranes were formed from the synthesis of nanosheets from the *meta*-bromination of the same molecule. In the process, an ANGM with a pore size below 1 nm can be controlled by altering the number of triple bonds between two phenyl rings as shown in Figures 7A–D. Four ANGM-n (*n* = 1, 2, 3, and 4) membranes family and three *γ*-graphyne family membranes were investigated. The MD simulation procedures employed were already established in reported literatures (Lin and Buehler, 2013; Xue et al., 2013; Zhu et al., 2013; Kou et al., 2014; Mehrdad and Moosavi, 2019). LAMMPS package (Plimpton, 1995) was used for all MD simulations analysis with Visual Molecular Dynamics (VMD) program (Humphrey et al., 1996). The result obtained by investigating the effect of external pressure as shown in Figure 7E, reveal that a linear relationship exists between the water flux and pressure, and the slope represent the membrane permeability. The order of the water flux performance are graphyne-5 *>* ANGM-4 *>* graphyne-4 *>* ANGM-3 *>* ANGM-2 *>* graphyne-3 *>* ANGM-1. Furthermore, the effect of external pressure on ion-rejection performance was also examined (Figure 7F). A slight decrease was observed in the ion rejection of all the membranes with the exception of graphyne-5. At pressure ≤150 MPa, graphyne-5 membrane exhibits a sharp decline but later increase with increasing pressure above 150 MPa. This action may be due to the rapid movement of water molecules through the membrane at high pressure, which consequently limits the movement of ions across the membrane. Previous studies have reported graphyne-3 membrane as the best membrane for desalination (Lin and Buehler, 2013; Xue et al., 2013; Kou et al., 2014), but recent studies revealed that ANGM-2 and ANGM-3 outperformed graphyne-3 membrane with higher permeabilities and 100% ions rejections. This outstanding performance hence, makes ANGM-2 and ANGM-3 membranes preferred options than graphyne-3 membrane in water desalination process.

### 4.2 Functionalized graphyne membrane for water desalination

Most preceding studies have largely used MD simulations to investigate the desalination potential of bare graphyne membranes. However, in real water desalination process or contaminated wastewater separation, these bare graphynes usually get functionalized by protons or hydroxyl radicals present in water (Psofogiannakis and Froudakis, 2012). Therefore, functionalization study is essential for its seawater desalination potential application. To address this, Raju et al. (2018) investigated the desalination performance of bare and hydrogenated (H) *α*-graphyne and *γ*-{2,3,4}-graphyne membranes using molecular dynamics simulations and upscale continuum analysis. Their results show that water flux through the membranes reduce as a function of applied external pressures with graphyne-4 and H graphyne-4 having the highest permeability. The hydrogenated *γ*-2-graphyne membrane has the lowest pore size and does not allow the passage of water for pressures lower than 100 MPa. Meanwhile, for the salt rejection, all the membrane tested have rejection values above 75% for pressure up to 2 GPa, with the exception of graphyne-4. Although, *γ*-graphyne-{2,3}, H *γ*-graphyne-3, α-graphyne and H α-graphyne membranes yielded high water fluxes, they were able to reject more than 90% of the ions for pressures up to 1 GPa. Moreover, upscale continuum analysis was used to complement the atomistic scale investigations. The analysis showed that the significant increase in permeability in the MD simulations did not match the real-life RO system because of some transport limitations but predict that graphyne membrane higher flux can permit about six times permeate recovery. It was further observed that salt rejection decreases with increasing pore size and applied pressure. The H membranes showed improved salt rejection performance compared to the bare ones owing to their lower pore area. Although, that was achieved with compromised water flux. Their study affirms that pore functionalization is an important factor to be considered while evaluating membranes for desalination.

**FIGURE 8:**
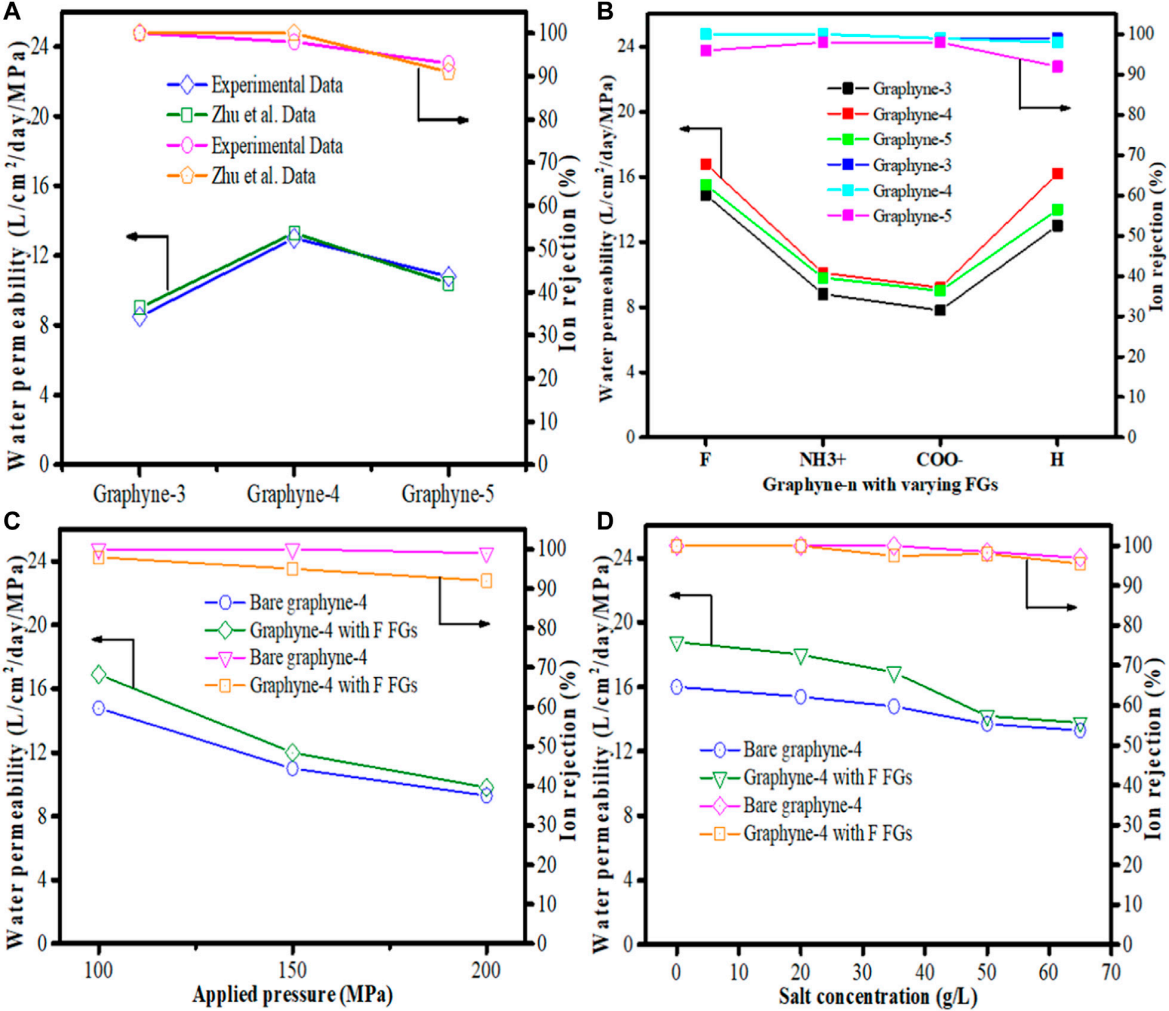
Water permeability and ion rejection for **(A)** graphyne-n (*n* = 3,4,5), Zhu et al. (2013) **(B)** graphyne-n (*n* = 3,4,5) with functional groups tested at 100 MPa and 35 g/L **(C)** bare graphyne-4 and “–F” functionalized graphyne-4 at applied pressures of 100, 150, and 200 Mpa at salt concentration of 35 g/L. **(D)** Bare graphyne-4 and “–F” functionalized graphyne-4 tested at salt concentration of 0, 20, 35, 50, 65 g/L at applied pressure of 100 Mpa (Adapted from Mehrdad and Moosavi, 2019).

In real-life desalination systems, carbon atoms are not fixed. However, most of the MD simulation studies applied in graphyne membrane desalination have fixed carbon atoms in the membrane to prevent out-of-plane displacement. Mehrdad and Moosavi (2019), therefore solves this problem by investigating the water permeability *via* three monolayer graphyne-n (*n* = 3, 4, and 5) membranes using different functional groups such as hydrogen (–H), fluorine (–F), carboxyl (COO^−^) and amine (NH_3_^+^) introduced into their pores. This they claim will aid the bending out of plane of the functionalized groups which in turn will align with water flow and increase the effective diameter of the pores as also supported by Raju et al. (2018). The MD simulation procedures used have been documented (Lin and Buehler, 2013; Cohen-Tanugi and Grossman, 2014), however, it is important to note that Lennard-Jones (LJ) parameter was used to model non-bonding interactions. Outcome from the study shows that water permeability through graphyne-4 was higher than graphyne-5 membrane (Figure 8A) because of the higher distribution density of nanopores on graphyne-4. This agrees with the work conducted by Zhu et al. (2013). In addition, the effect of the functional groups applied to the pores of all the graphynes-n (*n* = 3, 4, and 5) were examined. Results from the investigation showed that water permeabilities exhibited a dual nature. Charge distribution on the “–F” and “–H” functionalized graphynes membranes increase their water permeabilities while in NH_3_^+^ and COO- functionalized graphynes membranes, the water permeabilities decreased. Meanwhile, for each membrane, graphyne-3 and graphyne-4, ions rejection performance improved (Figure 8B). Optimally performing membranes (bare graphyne-4 and “–F” functionalized graphyne-4), were selected to investigate the influence of applied pressure. At increased pressure values, a corresponding decrease in water permeabilities was observed, although, “–F” functionalized graphyne-4 performs better than bare graphyne-4 membrane at all applied pressures (Figure 8C). Another attempt was made to examine the influence of salt concentration on the system performance using bare and F functionalized graphyne-4 membranes, respectively at 100 MPa. As shown in Figure 8D, there was decline in both the water permeability and the ion rejection of the membranes as salt concentration increases. However, in all, “–F” functionalized graphyne-4 membrane outperformed the bare graphyne-4 and other graphyne membranes considered thus, making it the best candidate for desalination.

**FIGURE 9:**
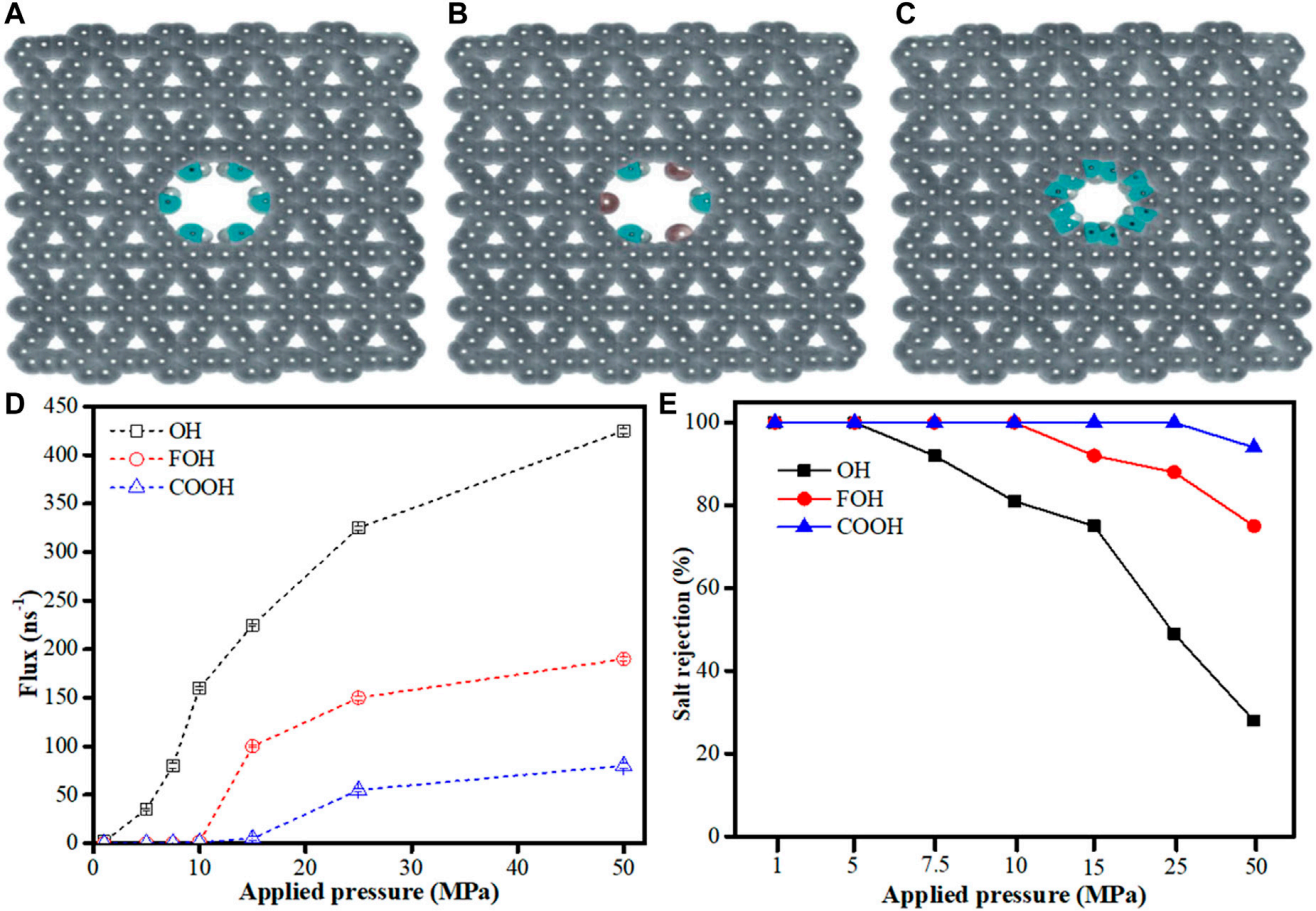
Functionalization *γ*-graphyne-1 membrane systems on the pore edge by **(A)** hydroxyl (-OH), **(B)** fluorine and hydroxyl (FOH) and **(C)** carboxylic acid (-COOH). **(D)** The water flux across the functionalized pores of the three *γ*-graphyne-1 membranes. **(E)** The salt rejection of functionalized pores at various applied pressures (Adapted from Azamat et al., 2020).

To gain better understanding of graphyne membranes pore functionalization and their influence on water desalting, Azamat et al. (2020) investigated the potential of functionalized *γ*-graphyne membrane pores and their allotropes for water desalination process. They employed MD simulation technique to examine one-atom-thick *γ*-graphyne-1 nanosheets with different functional groups on their pore edges. Three functional groups namely hydroxyl (–OH), fluorine (–F), and carboxylic acid (–COOH) were used to create the functionalized *γ*-graphyne-1 membranes as shown in Figure 9A–C. DFT was used to optimize the functionalized graphynes using the B3LYP theory level with the aid of GAMESS software (Schmidt et al., 1993) in order to obtain partial charges of all atoms. NAMD 2.18 and VMD 1.9.3 software were both used for all the MD runs and analyses (Humphrey et al., 1996; Phillips et al., 2005). The result obtained by investigating the effect of applied pressure showed that the water flux moving through the membrane pores increased with increasing pressure and the water flux of–OH functionalized *γ*-graphyne-1 membrane was higher than other functionalized *γ*-graphyne-1 membranes studied (Figure 9D). Meanwhile, at the absence of applied pressure, the water flux was zero. Furthermore, at low pressures (*<*7.5 MPa), Na^+^ and Cl^−^ions could not penetrate all the functionalized *γ*-graphyne-1 membranes, thereby having salt rejection of 100%. However, few ions could pass at higher applied pressure with corresponding reduction in salt rejection especially in–OH functionalized *γ*-graphyne-1 membrane. On the other hand, –COOH functionalized *γ*-graphyne-1 membrane yielded appreciable salt rejection at high applied pressure but has low permeability (Figure 9E). All the same, these functionalized *γ*-graphyne-1 membranes can better serve as effective tools for water desalination with high water flux and high salt rejection.

In addition to previously reported studies, a novel GFM was recently adapted by Jahangirzadeh et al. (2022) to explore their potential for water desalination. The group applied functionalized graphenylene nanosheet membrane for the first time for water desalination using MD simulations, although, it has previously been used for gas separation (Wang et al., 2020). A graphenylene nanosheet was prepared and functionalized with Fluorine (–F), hydrogen (H), combined fluorine & hydroxyl (–F & -OH), and combined fluorine & hydrogen (F & H) at their pore edges. The MD simulation procedures used followed those employed by Azamat et al. (2020). Undertaken at different pressures from 5–100 MPa, their results indicate that water molecules passing per 1 ns for each of the four functionalized graphenylene membrane increased with increasing pressure. The water permeability of the membranes was examined with respect to the applied pressure. Interestingly, graphenylene membrane functionalized with -F and combined “–F” & “-OH” exhibited the highest permeabilities achieved at 10 and 5 MPa, respectively. Although when compared, graphenylene membrane functionalized with “–F” & “-OH” has the highest permeability due to the combined hydrophilicity nature of the two membranes. On the other hand, membrane functionalized with “–H” has the lowest permeability because of the hydrophobic nature of “–H” atoms, which affects its hydrogenated pore. Moreover, the percentage salt rejection decreases consistently with increasing pressure. In contrast to the performance of the membrane permeability, graphenylene membrane functionalized with H exhibited the highest salt rejection percentage. Although, the percentage of salt rejection by membranes functionalized with -F and combined “–F” & “-OH” at 10 MPa and 100 MPa can reach up to 99.4% and 84.5%, respectively. Meanwhile, at pressure <10 MPa, all the functionalized graphenylene membranes had perfect salt rejections of 100%, suggesting their potentials for water desalination.

### 4.3 Ethanol-water separation and purification

The separation and purification of ethanol–water liquid mixture is an important process in bioethanol production and other chemical industries that deal with biofuels derived from biomass (Nalaparaju et al., 2011). Although in limited accounts, application of GFM membranes has been extended beyond water-salt separation and desalination to separation of ethanol-water mixtures. These GFM membranes could effectively purify the mixtures because of the steep energy barriers of penetration that ethanol molecules usually encounter during separation (Yeo et al., 2019).

**FIGURE 10:**
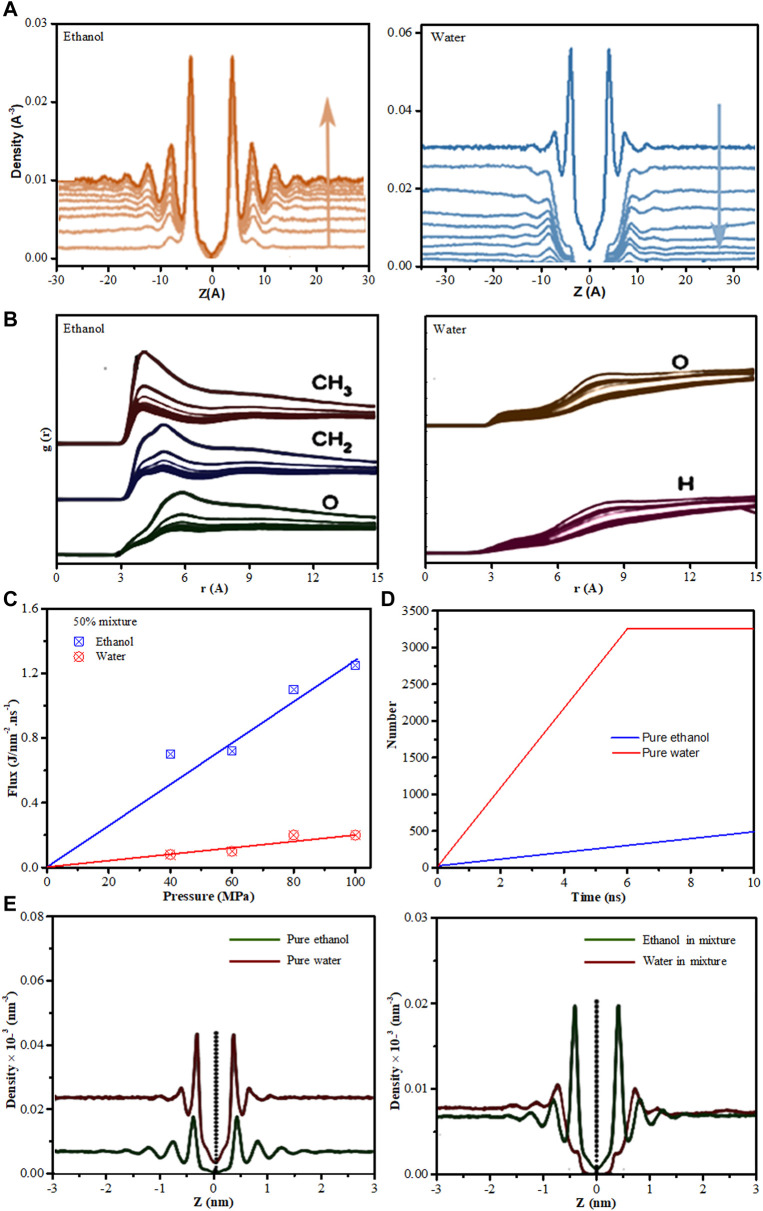
**(A)** Density profiles for ethanol and water on the graphyne-4 surface for ethanol concentration of 10%–90% (mole fraction) with the pure solvents (given in the black line). The direction of blue arrow represents the increase of ethanol concentration. The reference *z* = 0 corresponds to the membrane position. **(B)** RDF for the graphyne-4 carbon atoms with the carbon atoms of CH_3_– and CH_2_– and also the oxygen atoms in ethanol; and with oxygen atoms and hydrogen atoms in water, in different ethanol compositions (from 10 mol% to 90 mol%). **(C)** Fluxes of water and ethanol as a function of the applied pressures. **(D)** The filtered molecular number as a function of simulation time for pure water and pure ethanol through graphyne-4 membrane under 100 MPa. **(E)** Density distributions along *z*-axis for pure water and pure ethanol (left) and for the 50 mol% mixture (right) on the graphyne-4 surface [Figures **(A**,**B)** were adapted from Liu et al., 2016 while **(C**–**E)** were adapted from Zhang et al., 2019].

Liu et al. (2016) *via* MD simulations investigated the interfacial adsorption behavior of ethanol-water mixture near the surfaces of graphyne-n sheets with *n* = 3, 4, and 5. All MD simulations were carried out using LAMMPS package (Plimpton, 1995). Water was modelled by the SPC/E model (Berendsen et al., 1987) while ethanol was modeled based on OPLS-AA force field (Fern et al., 2007; Metya et al., 2014). The intermolecular interactions were described by Lennard-Jones (L-J) 12–6 potential and Coulombic interaction. Other parameters used such as graphynes bond lengths and lattice were obtained from Narita and Nagai (1998). By investigating the interfacial structural properties of ethanol-water mixtures near the single-layer graphyne sheet, the density profile of ethanol and water, for ethanol concentration of 10%–90% in mole fraction, and that of pure solvent using graphyne-4 was presented (Figure 10A). It was observed that the graphyne sheet stimulated long-range ordered interfacial distribution of ethanol with the density profiles displaying sharp adsorption peaks with few lower peaks. Meanwhile, the water density profile near the surface shows a quick decline, hence forming a water depletion region. They reported similar interfacial density profiles behavior for ethanol-water mixtures near the surfaces of graphyne-3 and graphyne-5. Radial distribution function also confirmed the preferential contact of ethanol molecules with the graphyne surfaces as there are obvious peaks for ethanol near the surfaces, which were absent for water. Methyl carbon were found to be closer to graphyne carbon in RDF peak positions than that of oxygen atoms in ethanol (Figure 10B). This ethanol preferential adsorption over water connotes the micro-phase demixing or separation for ethanol-water mixtures near the graphyne surface. This demixing behavior was more pronounced in graphyne-3 surface because of the decrease in pore area compare to graphyne-4 and graphyne-5 surfaces as their nanopores predominantly occupied the ethanol molecules from the mixture. This behavior shows the strong hydrophobic interaction between amphiphilic ethanol molecules and graphyne carbon surfaces, hence making polyporous graphyne surfaces a potential two-dimensional separation membrane (Liu et al., 2016).

Similar study on ethanol/water mixtures separation using two-dimensional (2D) nanoweb graphynes was carried out by the same group (Zhang et al., 2019). They employed MD simulations to computationally investigate the permeation performance of liquid ethanol–water mixtures across polyporous 2D *γ*-graphyne sheets. The MD simulation utilized followed same procedures as their initial study (Liu et al., 2016). The flux of ethanol–water mixture through graphyne-4 membrane under various external hydrostatic pressures were examined and their results showed that the flux of ethanol in the mixture is higher than that of water (Figure 10C). Meanwhile, the permeation carried out for individual species over time showed that water permeate faster through the membrane than pure ethanol (Figure 10D). This behavior elucidate the competitive and selective permeation of ethanol relative to water. Furthermore, the interfacial density profiles analysis was done to further understand the permeation performances. A favorable adsorption of ethanol relative to water, on graphyne-4 membrane surface in the ethanol–water mixture with significant adsorption peak for ethanol molecules was observed (Figure 10E). This further strengthen the assertion that amphiphilic ethanol molecules are able to predominantly concentrate/adsorb on hydrophobic carbon surfaces. This is in agreement with reported experimental (Severin et al., 2014), and theoretical results (Metya et al., 2014; Zhao and Yang, 2015).

To gain further understanding of the interfacial adsorption and permeation of ethanol–water mixtures on graphynes, the group (Zhang et al., 2019), combined dispersion-corrected density functional theory (DFT-D) and classical MD simulation strategy was utilized. Force fields such as Amber, OPLS and Charm have been used to represent the interfacial interaction of graphynes (Kou et al., 2013; Lin and Buehler, 2013; Liu et al., 2016), however, these methods preferentially use sp^2^ carbon parameters to represent graphyne atoms. As graphyne has sp–sp^2^ hybridized carbon atoms, the authors believes that adopting sp^2^ instead of sp–sp^2^ could lead to underestimation of the interaction between molecules and graphyne, which in turn will lead to bias description of interfacial behavior near graphyne surfaces. Therefore, the group (Zhang et al., 2019), utilized sp–sp^2^ hybridized carbon atoms to cater for this bias. As such, DFT-based quantum computation was initially applied to identify the nature of the interaction of ethanol and water on graphyne-n by parameterizing force field potential, thereafter, MD simulation was used to probe the interfacial adsorption and permeation properties of ethanol–water mixtures on the graphyne surfaces. Similar to their previous study (Liu et al., 2016), the acquired density profile of ethanol display a sharp adsorption peak near the graphyne surface, which represent a strong adsorption behavior while that of water rapidly deplete near the graphyne surface. Furthermore, the permeation of the ethanol–water mixture (50 mol%) displayed considerable ethanol molecules permeation through the graphyne membranes (especially graphyne-4 pores), hence demonstrating the enrichment of ethanol on the permeate side. Furthermore, the adsorption densities on the graphyne surface using the DFT-based force field displayed stronger adsorption densities compared to the OPLS force field. This observation thus, confirms the underestimation of the binding affinity between molecules and graphyne, which has brought about the misinterpretation of the interfacial properties.

Bringing all these three studies on ethanol-water mixtures separation together, it can be inferred that, the energy barrier for each of the graphyne sheet differs depending on the orientation of the ethanol molecule relative to the pore. Additionally, the dispersion attraction of ethanol molecules was stronger as they adsorbed more to the grapyhne surfaces than water molecules. Amongst the three graphyne membrane sheet tested i.e., graphyne-3, graphyne-4 and graphyne-5, graphyne-4 membrane tends to exhibit the most desirable performance for separation of water from ethanol.

## 5 Challenges and outlooks

The recent progress on the works carried out on GFM *in lieu* of its application in water purification and desalination have been summarized, and most authors acknowledged the inherent potentials of GFM membranes in serving as ideal candidates and alternative to commercial separation membrane currently used for water desalting. However, these potentials remain latent because of some present and near future challenges such as lack of lab-scale GFM membrane fabrication, diversity in theoretical and computational analysis and desirable rejection of heavy metal/organic rejection contaminants.

### 5.1 Lack of lab-scale GFM membrane fabrication

It is no doubt that GFM-based membranes are still in their emerging stage of development. There are still several hurdles to cross and one of such big hurdles is to be able to successfully synthesize them at lab-scale. At present, the theoretical and computational research works carried out on the application of GFM membranes for water desalination cannot be adjudge successful until a sure pathway of preparing these membranes, first in the laboratory and eventually at scalable and economical manufacturing process. There is need to extend the characterization of these membranes beyond theoretical and computational predictions into concrete experimental validations. One major factor of consideration in this direction is the quality of GFM material to be used. Although, the synthesis and properties of GFMs have been reported in literatures (Jia et al., 2017; Gao et al., 2019; Kang et al., 2019; Gong et al., 2020), some shortcomings in synthesized GFMs such as defects in the form of wrinkles, tears and vacancies needs to be addressed if it would feature in membrane application. In principle, a defected material would adversely affect the separation performance of the membrane, and most especially the selectivity (Favre, 2022). Therefore, it is essential that a non-destructive approach is exploited. Moreover, the lab-scale prepared GFM-based membranes must possess attractive properties such as high thermal and mechanical strength so that they can withstand high loading pressure during separation applications.

### 5.2 Diversity in theoretical and computational analysis

Largely, all the investigations carried out to examine the application of graphyne as membrane for water desalination and purification utilized MD simulations. Researchers and research teams were at liberty to choose which computational models and parameter to employ for their works depending on their experimental and system set-up. This resulted in inconsistency in the theoretical and computational determination of the GFM membrane permeabilities and selectivity. The downside of this approach was that, it resulted in inconsistency and variation in the overall theoretical outcome presented in those studies. For instance, some MD computational studies established through their findings (Xue et al., 2013; Kou et al., 2014) that graphyne-3 membrane can achieve 100% salt rejection at higher pressure, which is not attainable with graphyne-4 and graphyne-5 membranes, even though they have higher permeabilities than graphyne-3 membrane because of their larger pores. However, the study conducted by Zhu et al. (2013) argued otherwise that graphyne-4 can also achieve 100% salt rejection even at higher pressure even with the advantage of high permeability, which then made it a better choice over graphyne-3 membrane for water desalination application as claimed in the study. This disagreement could have resulted from factors such as water model type used, force field parameters selected, pressure loading strategy adopted and so on. Another issue with the application of the theoretical and computational methods is how accurate and reliable the reported permeabilities and rejections are. DFT are useful in estimating the interactions between molecules with non-negligible quantum effect role. It uses functions to estimate the electron correlation and exchange energies so as to forecast electron density of atomic systems (Cohen-Tanugi and Grossman, 2015). In theory and computation studies of membrane water desalination, DFT studies in relation to energy landscape could be used to predict very high selectivity, although, this is usually achieved without given due consideration to the influence of some parameters such as molecule population, temperature and pressure fluctuations in real-life systems. Meanwhile, MD simulations can be used to achieve better permeability depending on the number of permeation events of desired species (Qiu et al., 2019). Therefore, to address the inconsistency issue, it is vital to combine and engage both DFT and MD methods simultaneously in a computational study of water desalination through GFMs, to be able to understand how each parameter and model affect the predicted performance. In addition, more studies should focus on combining QM and MD (i.e., hybrid quantum mechanics), to ascertain the previously established performance of the GFM membranes.

### 5.3 Desirable rejection of heavy metal/organic contaminants

One of the successes of conventional and lab-scale prepared RO, NF and membrane distillation membranes is seen in the efficient separation of heavy metal ions from contaminated water resources (Abass et al., 2016; Qasem et al., 2021; Lasisi et al., 2022) and organic solvents separation (Chen et al., 2019; Dai et al., 2019). Interestingly, their removal efficiency can reach up to 98.75% and 99.3% (Ozaki et al., 2002). In addition, pure and functionalized nanoporous graphenes have been employed as RO membrane either by experimental or MD simulations to examine the separation performance of heavy metal ions (Park et al., 2016; Li et al., 2017), and excellent separation were realized. However, this process (especially heavy metal/organic contaminant rejection by MD simulations) is not only limited to graphene sheets, Lin and Buehler (2013) used pure graphyne membrane to remove heavy metal/organic contaminants from wastewater and seawater *via* MD simulations. The membrane showed higher rejection for CuSO_4_ and NaCl compared to C_6_H_6_ and CCl_4,_ which were directly linked to their different interaction strengths with water molecules. Notwithstanding, the separation performance was not comparable to those of nanoporous graphenes and as-prepared RO, MD, and NF membranes previously reported. Generally, the ion hydration of heavy metal ions is very strong due to their enlarged Coulombic interaction, and this usually results in weak dehydration effect when passing through the graphene/graphyne pores (Ferchmin, 2002) hence, a low heavy metal ions rejection is observed. One way to address this issue is through membrane functionalization. Functionalized graphene sheets have been prepared experimentally (Mishra and Ramaprabhu, 2011) and theoretically (Li et al., 2017) and used to remove high concentrations of metal salt ions. Their findings show that heavy metal ions could gain improved interaction with the pore edge of the functionalized graphene, which in turn influence the ion rejection performance. This same principle of functionalization can be applied for GFM sheets to improve their heavy metal ions rejection performance. Beside the work of Lin and Buehler (2013), which reported the application of bare graphyne membrane, no other study has considered heavy metal ions rejection performance *via* functionalized graphyne membranes, or even establish further the validity of bare graphyne membranes heavy metal ions rejection performance till date. Therefore, more studies that will investigate the separation performance of functionalized graphynes pores for heavy metal ions, and achieve desirable outcomes that are comparable to conventional and as-prepared RO and NF membranes are highly necessary. Besides, a thorough understanding of the molecule’s behavior inside the graphyne nanopores is also necessary as mass transport *via* pure graphyne pores is governed by molecular sieving mechanism. This knowledge will help membrane designers to make right selection of graphyne types and chemical functionalization that will meet the needed separation demand.

## 6 Conclusion

In this review, the different theoretical and computational progresses made in the research of GFMs for water purification and desalination is investigated. In the first instance, the mechanical, structural, electrical conductivity, and thermal properties of graphynes, graphdiynes, and other GFMs were analyzed for their suitability in water desalination. In addition, systematic evaluation of the different computational approaches engaged in water desalination studies were provided. Outcomes of reviewed literature on GFM membranes demonstrated their extraordinary performance in water purification and desalination. For instance, *via* MD simulations, graphyne-3 and graphyne-4 are reported to achieve permeability up to approximately two orders of magnitude greater than the current best-performed RO membranes experimental values, while simultaneously maintaining almost a complete 100% salt rejection. The interfacial adsorption behavior of liquid ethanol–water mixtures near the surfaces of GFM membrane sheets and their permeation performance were also examined. Although, few reports have published these findings, it was observed that graphyne-4 membrane amidst others exhibited the most desirable performance for separation of water from ethanol, and shows favorable adsorption of ethanol relative to water in the ethanol–water mixture. Moreover, efforts are currently being made in improving the separation performance of these membranes *via* chemical functionalization of their pore edges. Inarguably, some notable efforts have been made in the efficient application of GFMs in water purification and desalination. However, there are challenges that need to be surmounted in order to make GFM materials more attractive. Approaches that can be exploited include translation of computational studies into lab-scale experimental tool, then subsequently into industrial scale application, optimizing theoretical and computational tools to achieve consistent outcomes, and establishing basic understanding of molecules transport behavior inside graphyne nanopores to achieve improved permeability and selectivity performance. Interestingly, GFMs are endowed with unique pore characteristics that can make them ideal candidates for future water desalination. This work opens up window opportunities to explore new and emerging research strategies/pathways to overcome challenges associated with practical application of GFM membranes, which are still largely in their early stage of development.
